# Exogenous Administration of Recombinant MIF at Physiological Concentrations Failed to Attenuate Infarct Size in a Langendorff Perfused Isolated Mouse Heart Model

**DOI:** 10.1007/s10557-016-6673-2

**Published:** 2016-06-22

**Authors:** Xavier Rossello, Niall Burke, Christian Stoppe, Jurgen Bernhagen, Sean M. Davidson, Derek M. Yellon

**Affiliations:** 1 The Hatter Cardiovascular Institute, University College London, 67 Chenies Mews, London, WC1E 6HX 2 UK; 2 Department of Intensive Care Medicine, University Hospital, Aachen, RWTH Germany; 3 Vascular Biology, Institute for Stroke and Dementia Research, Klinikum der Universität München, Ludwig-Maximilians-University, 81377 Munich, Germany; 4 Munich Cluster for Systems Neurology (SyNergy), 81377 Munich, Germany; 5 NIHR UCLH Biomedical Research Centre, University College London Hospital & Medical School, London, UK

**Keywords:** Ischemic preconditioning, Ischemia–reperfusion, Myocardial infarction, Macrophage migration inhibitory factor

## Abstract

**Purpose:**

Evidence suggests a two-pronged role of endogenous macrophage migration inhibitory factor (MIF) release in ischemia/reperfusion injury. We aimed to assess whether its exogenous administration confers cardioprotection.

**Methods:**

Male C57/BL6 mice were randomly allocated to receive recombinant mouse MIF (rMIF) at physiological (ng/mL) concentrations in a dose–response fashion before or after a protocol of 35 min of ischemia and 2 h of reperfusion in an isolated Langendorff-perfused model with infarct size as endpoint. Isolated primary cardiomyocytes were also used for cell survival studies using rMIF at a supra-physiological concentration of 1 μg/mL. Pro-survival kinase activation was also studied using Western blot analyses.

**Results:**

Exogenous MIF did not elicit a cardioprotective effect either when administered before the ischemic insult or when applied at reperfusion. rMIF did not confer protection when it was applied immediately before or after a hypoxia/reoxygenation insult in primary isolated cardiomyocytes. Consistently, hearts treated with MIF did not show a significant increase in phosphorylated Akt and ERK1/2.

**Conclusion:**

The exogenous administration of rMIF in a physiological concentration range both before ischemia and at reperfusion did not show cardioprotective effects. Although these results do not address the role of endogenous MIF after an ischemic insult followed by reperfusion, they may limit the potential translational value of rMIF.

## Background

Acute myocardial infarction (AMI) is a leading cause of mortality and morbidity worldwide. Early coronary reperfusion limits infarct size (IS) and improves prognosis. Paradoxically, reperfusion itself induces additional damage to the myocardium, known as ischemia/reperfusion injury (IRI) [1]. Further IS-limiting therapies beyond early reperfusion are needed, since IS is the main prognostic determinant in post-AMI patients [2].

Macrophage migration inhibitory factor (MIF) is a pleiotropic inflammatory cytokine with chemokine-like functions [3], but also cell-intrinsic roles in cell stress and survival. MIF is expressed by cardiomyocytes in the heart, and its secretion from the heart is triggered after an ischemia–reperfusion insult [4, 5], potentially affecting myocardial function [6].

Endogenous MIF has emerged as a key player in cardioprotection of myocardial IRI [7]. Its cytoprotective role has been mainly studied in *Mif*-deficient mice, demonstrating that the lack of this cytokine increases the injury caused after an ischemic insult followed by reperfusion, whilst exogenous MIF administration in those knockout (KO) mice restores the amount of injury to that observed in the wild types (WT). Endogenous MIF appears to confer cardioprotection through several mechanisms: 1) AMPK activation through interaction with the cell surface receptor CD74, promoting glucose uptake in the heart [4]; 2) inhibition of JNK-mediated apoptosis [8]; and 3) MIF’s own antioxidant properties [9], further increased with a S-nitros(yl)ation modification of MIF at cysteine 81 residue [10].

MIF also activates PI3 kinase in different cell types [11–13]. It is well known that Akt and ERK act as survival proteins and form part of the well described Reperfusion Injury Salvage Kinase (RISK) pathway [1]. Activation of this signaling cascade has been associated with protection from I/R injury by both the conditioning phenomenon and a range of pharmacological agents. As such our hypothesis proposes that MIF by activating PI3 kinase and ERK1/2 would have a beneficial effect on the heart following IRI.

Preliminary data indicate that MIF is involved in the molecular mechanisms of anaesthetic induced preconditioning [14]. Even small-molecule MIF agonists enhancing MIF-mediated AMPK-activation have been developed as a potential therapeutic drug [15]. However, whilst the administration of recombinant MIF in a murine in vivo myocardial I/R setting reversed the increased infarct size seen in *Mif*-deficient mice [10], the cardioprotective efficacy of MIF administered in animals without genetic manipulation has not been elucidated. The confirmation of an IS-limiting effect after an exogenous MIF administration would have a significant translational value.

While some clinical findings showed MIF’s organoprotective effects in patients exposed to myocardial I/R following cardiac surgery [16], some in vivo and clinical studies have suggested deleterious properties of MIF release after an AMI. For example, elevated MIF blood levels after an AMI have been positively correlated with IS and negatively associated with the extent of cardiac remodeling [17]. In addition, as MIF is a cytokine playing an important role in the inflammatory response that occurs early after AMI [6], it has been suggested that *Mif* deficiency confers cardioprotection from prolonged IRI by suppressing these responses [9, 18], whilst it actually does the contrary effect after a shorter ischemic insult. These paradoxical results are potentially explainable, because the inflammatory response is a prerequisite for adequate healing and scar formation, that may become adverse if persistent [19]. Moreover, in other settings where there is significant MIF release, such as in sepsis, MIF has shown cardio-depressant properties [20, 21].

All this evidence points towards to a two-pronged role of endogenous MIF, although the key question, with further translational value, of whether its exogenous administration is cardioprotective when applied to wild-type animals remains unanswered. Hence, the aims of this study were (1) to analyze the effect of the exogenous administration of MIF on IS in a dose–response fashion, using physiological concentrations of MIF, either applied before or after an ischemic insult, (2) to analyze whether a very high dose of MIF confers greater protection at cardiomyocyte level, and (3) to study the RISK signaling pathway activation after the exogenous administration of MIF.

## Methods and Materials

### Animals and Chemicals

Animals used were male C57BL/6 mice (9–12 weeks, 24–28 g weight). Recombinant mouse MIF (rMIF) and bradykinin were purchased from Biolegend (London, UK) and Sigma-Aldrich (Poole, UK), respectively. The bioactivity of rMIF was tested by the company using a bioassay that studied the migration of THP-1 cells in a trans-well experimental system. Doses of rMIF were chosen by evaluation of those applied in *Mif*-deficient mice in previous publications [8, 22] as well as its physiological plasma levels range [7, 17]. Bradykinin concentration dose administered at reperfusion was based on previous publications [23]. Dimethyl sulfoxide from BDH (Poole, UK) was used as the solvent for Bradykinin at a final concentration in the perfusion buffer of not more than 0.01 %.

### Study Design and Study Protocol

**Fig. 1:**
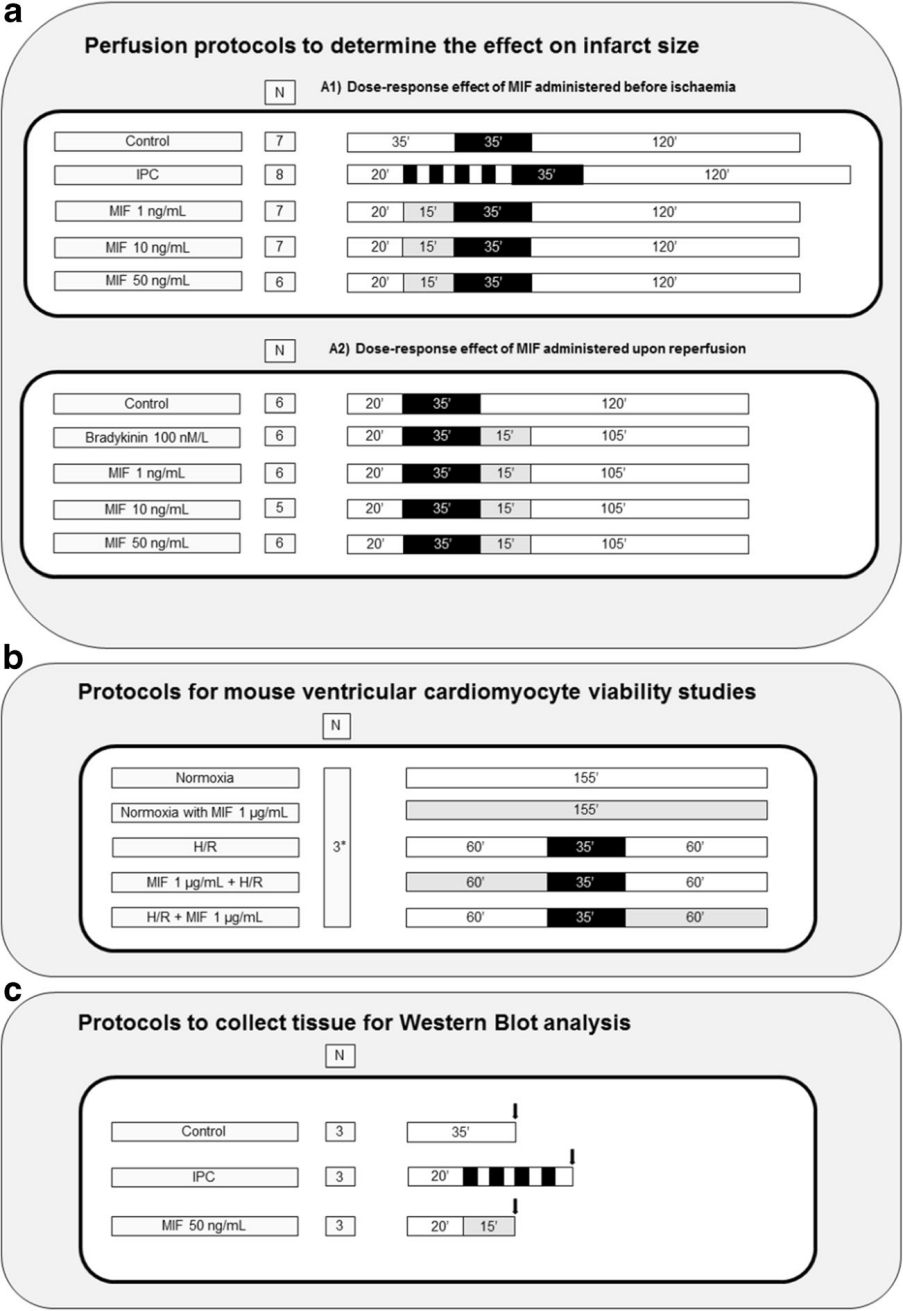
Study design and protocols. A black box represents a period of ischemia, whilst a white box represents a period of perfusion with Krebs–Henseleit buffer at 80 mmHg. A grey box represents the perfusion of MIF through the perfusion apparatus. The black arrows show the time point the samples for Western Blot analysis were taken. * In total, 3 animals were used to isolate cardiomyocytes and allocate them in 5 separate groups in each isolation. Abbreviations: H/R, hypoxia/reoxygenation; IPC, ischemic preconditioning; MIF, macrophage migration inhibitory factor

Four sequential sets of experiments were performed, requiring the use of 80 animals: 68 were allocated for perfusion experiment purposes (4 of them were excluded before randomization as they fulfilled pre-defined exclusion criteria), 3 were used for cardiomyocyte isolation, and finally 9 were used to collect samples for Western blot analyses. Hence, the study was divided into four consecutive parts, as illustrated in Fig. 1:

1. Effect on myocardial IS of the exogenous MIF administration before ischemia (Fig. 1a). Thirty-eight mice were allocated according to a randomized block design into the following groups: 1) Control; 2) ischemic preconditioning (IPC); 3) MIF 1 ng/mL; 4) MIF 10 ng/mL; and 5) MIF 50 ng/mL. IPC was induced by 4 cycles of 5 min global ischemia and 5 min reperfusion prior to index ischemia. MIF was applied 15 min before ischemia.
2. Effect on myocardial IS of the exogenous MIF administration upon reperfusion (Fig. 1a). Thirty mice were randomized into 5 groups to evaluate the effects of: 1) Control; 2) Bradykinin 100 nM/L; 3) MIF 1 ng/mL; 4) MIF 10 ng/mL; and 5) MIF 50 ng/mL. All MIF groups received the therapy immediately at reperfusion during 15 min.
3. Effect on percent of cell death of high-dose MIF administration in normoxia and hypoxia/reoxygenation (H/R) protocols in adult mouse cardiomyocytes. Three animals were used for cardiomyocyte isolation. Cardiomyocytes were randomly allocated into 5 groups (2 wells per group): (1) Normoxic control, (2) Normoxic MIF 1 μg/mL, (3) H/R control, (4) H/R with 1 μg/mL MIF before the hypoxic insult, and (5) H/R with 1 μg/mL MIF at reoxygenation.
4. Effect on ERK and Akt phosphorylation after the exogenous MIF administration in a Langendorff-perfused mouse heart model. Hearts destined for Western blot analyses were allocated in 3 groups: (1) Control, (2) IPC, and (3) MIF 50 ng/mL.

### Ex Vivo Isolated Langendorff-Perfused Mouse Heart Model of Acute Myocardial Infarction

Mice were given terminal anesthesia and anticoagulation with an intra-peritoneal injection of 60 mg/kg sodium pentobarbitone and 100 IU heparin, respectively. The heart was harvested and immediately submerged in ice-cold modified Krebs–Henseleit buffer (see below composition). The aorta was swiftly cannulated with a 21-gauge cannula and the heart retrogradely perfused on a murine Langendorff perfusion apparatus at 80 mmHg pressure.

Heart isolation and Langendorff perfusion were carried out with filtered modified Krebs–Henseleit buffer (composed of 118 mmol/L NaCl, 25 mmol/L NaHCO_3_, 11 mmol/L glucose, 4.7 mmol/L KCl, 1.22 mmol/L MgSO_4_.7H_2_0, 1.21 mmol/L KH_2_PO_4_, and 1.84 mmol/L CaCl_2_.2H_2_0) aerated with a mixture of O_2_ (95 %) and CO_2_ (5 %) in order to maintain pH at 7.42 ± 0.3, as previously described [24, 25]. After the assessment for exclusion criteria in an initial stabilization period of 20 min, each Langendorff perfused mouse heart was randomly assigned to one of the study protocols illustrated in Fig. 1a.

Pre-defined exclusion criteria were: 1) time between cessation of blood circulation and the start of perfusion in the Langendorff mode greater than 4 min, 2) temperature above or below the 37 ± 0.2 °C range, 3) flow rate of less than 1 mL/min or more than 6 mL/min on the Langendorff preparation during the stabilisation period.

All hearts were subjected to 35 min global ischemia and 2 h reperfusion prior to determination of IS by 2,3,5-triphenyltetrazolium chloride (TTC) staining.

### Myocardial Infarct Size Analysis

After global normothermic ischemia, IS was determined by injecting 5 mL of TTC in phosphate-buffered saline through the aortic cannula and incubating the heart for 10 min at 37 °C in order to demarcate the infarcted (white) versus viable (red) tissue [26]. After the incubation, the heart was weighed and then frozen overnight at −20 °C. Following this, it was sectioned perpendicular to the long axis and the slices transferred into 10 % neutral formalin buffer for 1 h.

Images were thereafter coded in order to blind the analyser. Planimetry analysis using Image J version 1.47 (NIH, Bethesda, MD) was carried out to accurately quantify the percentage IS in each heart as a proportion of the total heart volume.

### Isolation of Adult Mouse Cardiomyocytes

Ventricular cardiomyocytes were isolated from mouse hearts using Liberase digestion as described previously [27]. Briefly, hearts were excised and cannulated through the aorta before retrograde perfusion on a modified Langendorff system at 37 °C. Perfusion buffer consisted of NaCl 113 mmol/L, KCl 4.7 mmol/L, KH_2_PO_4_ 0.6 mmol/L, Na_2_HPO_4_ 0.6 mmol/L, MgSO4-7H2O 1.2 mmol/L, NaHCO3 12 mmol/L, KHCO3 10 mmol/L, Hepes Na salt 0.922 mmol/L, Taurine 30 mmol/L, 2,3-butanedione-monoxime 10 mmol/L, Glucose 5.5 mmol/L (pH 7.4). Following perfusion for 5 min to clear residual blood, enzymatic digestion was performed with a digestion buffer for 20 min (30 mL perfusion buffer with 5 mg Liberase (Roche, UK) and 12.5 μmol/L CaCl_2_). At the end of enzymatic digestion, both ventricles were isolated and gently disaggregated in 2 mL of digestion buffer. The resulting cell suspension was filtered through a 100 μm mesh and transferred for enzymatic inactivation to a tube with 10 mL of stopping buffer (perfusion buffer supplemented with fetal bovine serum (FBS) 10 % and Ca^2+^ was gradually re-introduced with three progressively increased CaCl_2_ concentration steps. Cells were then re-suspended in M199 (Invitrogen, UK) supplemented with L-Carnitine (2 mmol/L), Creatine (5 mmol/L, Taurine (5 mmol/L) and Penicillin (100 IU/ml), Streptomycin (100 IU/ml) and 25 μmol/L Blebbistatin.

### Experimental Protocol for Mouse Ventricular Cardiomyocyte Viability Studies

Prior to being subjected to induced hypoxia/reoxygenation, freshly isolated adult mouse cardiomyocytes were plated and stabilized for 1 h at 37 °C with M199 medium with the following supplements: creatine 5 mM, carnitine 2 mM, taurine 5 mM and penicillin/streptomycin (100 x). Cells were thereafter randomly allocated in each group and the medium was changed to normoxic buffer for 1 h, prior to being subjected to 30 min of hypoxia and 1 h of re-oxygenation to simulate IRI. MIF was administered in combination with normoxic buffer according to the protocol illustrated in Fig. 1b. Hypoxia was induced in an airtight hypoxic chamber using a buffer simulating the conditions of ischemia (in mM: 1.2 KH_2_PO_4_, 2.2 NaHCO_3_, 1.2 MgSO_4_, 127.8 NaCl, 14.8 KCl, 1 CaCl_2_ and 10 Na lactate, pH 6.4) bubbled with 95 % N_2_/5 % CO_2_. Re-oxygenation was achieved by replacing the buffer with the normoxic buffer (containing in mM: 1.2 KH_2_PO_4_, 22 NaHCO_3_, 1.2 MgSO_4_, 118 NaCl, 2.6 KCl, 1.2 CaCl_2_ 10.0 D-glucose; pH 7.4) bubbled with 95 % O2/5 % CO2. At the end of the re-oxygenation, the cells were stained with 1 mg/mL stock propidium iodide (PI) and imaged using a fluorescent microscope – red channel for PI and phase contrast for total cell population. The percentage of dead cells (as indicated by red fluorescence, PI-positive) in duplicate wells was calculated by fluorescence microscopy and was expressed as a percentage of the total number of cardiomyocytes.

### Western Blot Analysis

Hearts prepared for Western blot analysis were harvested following those protocols illustrated in Fig. 1c, and thereafter snap-frozen in liquid nitrogen. The tissue was then stored at −80 °C until further processing. The tissue was homogenized in protein lysis buffer, containing Tris pH 6.8 [100 nM], NaCl [300 Mm], NP40 0.5%pH, Halt protease inhibitor cocktail, Halt phosphatase inhibitor cocktail, 0.5 M EDTA and adjusted to pH 7.4. Homogenates were then briefly sonicated before being centrifugated at 4 °C to remove debris and DNA. Protein content was then determined using bicinchocinic aci (BCA) protein assay reagent (Sigma, UK) and protein levels corrected accordingly to ensure equal protein loading. NuPAGE LDS Sample Buffer (4X) (Thermofisher Scientific, UK) plus 5 % β-mercaptothanol were added and the samples were denaturated by heating to 90–100 °C for 10 min. NuPAGE Novex 10 % Bis-Tris protein gels (Thermofisher scientific, UK) were used for electrophoresis using the Mini Protean III system (Bio-Rad, UK). Gels were transferred onto immobilon-FL PVDF transfer membrane (MerckMillipore, UK) using wet transfer in a Bio-Rad Mini Trans-Blot. The membranes were blocked by incubating in 2.5 % bovin serum albumin/PBS tween and subsequently incubated with appropriate primary antibodies at 4 °C overnight. Primary antibodies used were acquired from Cell Signaling Technology: ERK1/2 (#9102), Phospho-ERK1/2 (Thr202/Tyr204) (#9101), Akt (#9272), Phospho-Akt (Ser473) (#9271). The day after, the membrane were probed with secondary antibodies with infrared fluorescent. Levels of protein were finally quantified using the Odyssey imaging system from Li-Cor Biosciences.

### Statistical Analyses

Images were coded in order to perform a blinded assessment of both IS and cell viability pictures. All values are presented at mean ± standard error of the mean. One-way ANOVA was carried out to compare percentages between groups, followed by post-test analysis using the Dunnett’s t-test for multiple comparisons when using a single reference category (ex vivo isolated Langendorff-perfused and Western Blot results) or by Bonferroni’s correction when performing multiple comparisons with different reference categories (studies of cell viability presented two separate controls: one within the normoxic group and another one within the H/R group). A two-sided *P*-value <0.05 was considered significant.

STATA software, version 13.1 (Stata Corp, College Station, TX, USA) and GraphPad Prism version 6.00 (GraphPad Software, La Jolla California, USA) were used to perform the analysis and the graphics. The results were reported according to the ARRIVE guidelines for reporting animal research [28].

## Results

### MIF before Ischemia–Reperfusion

**Fig. 2:**
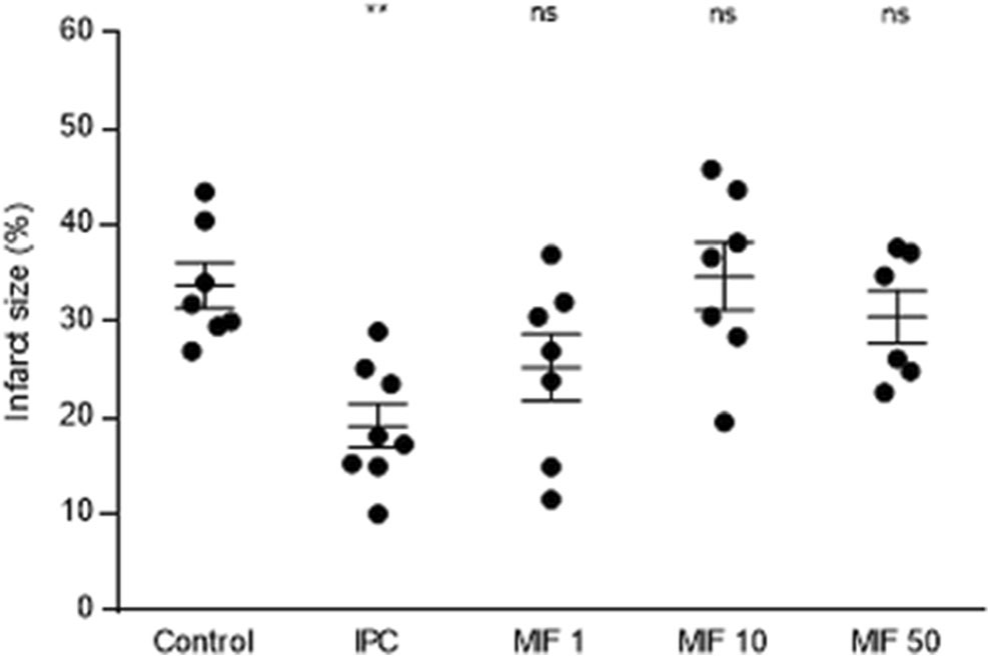
Dose–response effect of exogenous rMIF administered before ischemia on myocardial infarct size in the ex vivo isolated Langendorff-perfused mouse heart model (%). Bar graph showing the IS in all groups, expressed as mean ± SEM for infarct size (%), *n* = 6–8 per group. Overall *P*-value was 0.002, whilst in the post-hoc analysis, the control group was compared to IPC (**P = 0.003), MIF 1 ng/mL (*P* = 0.138), MIF 10 ng/mL (*P* = 0.998) and MIF 50 ng/mL (*P* = 0.859). Abbreviations: IPC, ischemic preconditioning; MIF, macrophage migration inhibitory factor

To determine whether rMIF is protective against IRI in mice when applied before ischemia, a dose response curve using the ex vivo isolated Langendorff-perfused mouse heart model was performed (Fig. 2). The IS after global ischemia was 31.8 ± 2.3 % in the control group, whilst the low dose of rMIF showed an IS reduction but without statistical significance (25.3 ± 3.5 %, *P* = 0.138 compared to control group). On the contrary, neither the mid-dose nor the high-dose administration of MIF showed any relevant difference in terms of IS (34.7 ± 3.5 %, *P* = 0.998; and 30.5 ± 2.8 %, *P* = 0.859, respectively). Finally, the application of a 4-cycle protocol of IPC, as a positive control, resulted in an infarct reduction of 17.7 ± 2.2 % (*P* = 0.003).

### MIF at Reperfusion

**Fig. 3:**
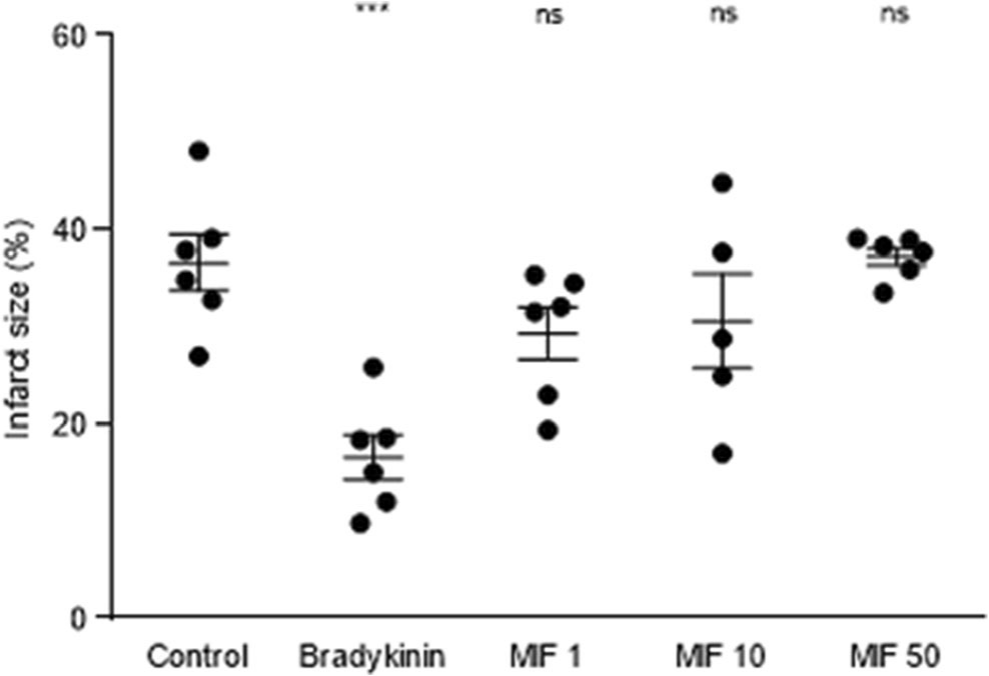
Dose–response effect of exogenous rMIF administered upon reperfusion on myocardial infarct size in the ex vivo isolated Langendorff-perfused mouse heart model (%). Bar graph showing the IS in all groups, expressed as mean ± SEM for infarct size (%), *n* = 5–6 per group. Overall *P*-value was <0.001, whilst in the post-hoc analysis, the control group was compared to bradykinin (***P < 0.001), MIF 1 ng/mL (*P* = 0.223), MIF 10 ng/mL (*P* = 0.412) and MIF 50 ng/mL (*P* = 0.998). Abbreviations: IPC, ischemic preconditioning; MIF, macrophage migration inhibitory factor

The exogenous administration of rMIF (1 ng/mL, 10 ng/mL and 50 ng/mL), applied upon restoration of coronary flow (reperfusion), did not confer any cardioprotective effect when compared to control group (IS of 36.6 ± 2.9 %) (Fig. 3). The low-dose of MIF did not show a significant reduction of IS (29.3 ± 2.7 %, *P* = 0.223). In the same vein, neither relevant effect was observed with the mid-dose (30.6 ± 4.9 %, *P* = 0.412) nor with the higher dose of MIF (37.2 ± 0.9, *P* = 0.998). A well-known cardioprotective agent was used as a positive control (bradykinin 100 nM), resulting in a highly significant IS reduction (16.6 ± 2.3, *P* = 0.001).

### MIF in Isolated Cardiomyocytes

**Fig. 4:**
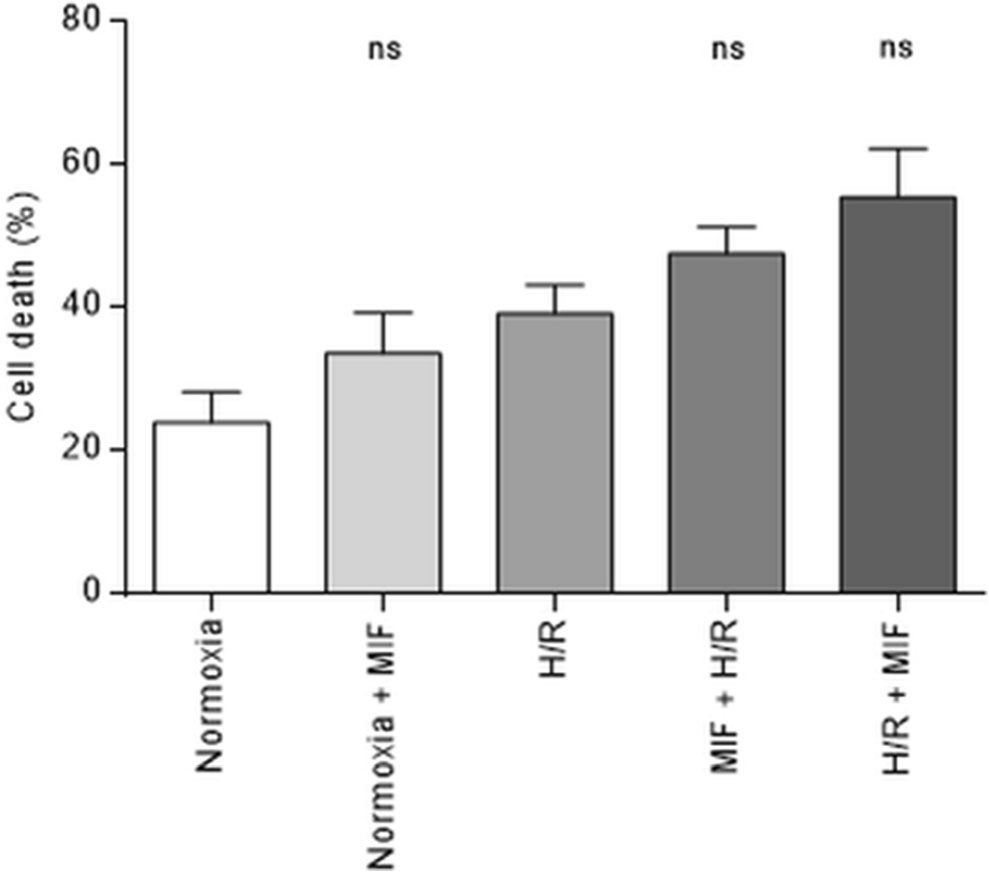
A supraphysiological concentration of rMIF does not increase survival of isolated adult cardiomyocytes in normoxic and H/R conditions. Bar graph showing the IS in all groups, expressed as mean ± SEM for infarct size (%), *n* = 6 wells per group coming from 3 animals. Overall *P*-value was 0.010. Comparisons between relevant groups did not show statistically significant differences: normoxia vs normoxia with rMIF 1 μg/mL (24.0 ± 4.25 % vs 33.7 ± 5.60 %, *P* = 0.610), H/R vs H/R with rMIF 1 μg/mL prior to the protocol (39.3 ± 3.94 % vs 47.6 ± 3.70 %, *P* = 0.794); and H/R vs H/R with rMIF 1 μg/mL after the protocol (39.3 ± 3.94 % vs 55.5 ± 6.85 %, *P* = 0.134). Abbreviations: H/R, hypoxia/reoxygenation; MIF, macrophage migration inhibitory factor

Given the unexpected results in the isolated Langendorff-perfused model, a supraphysiologic dose of MIF was applied in isolated cardiomyocytes without, before and after a protocol of H/R. Isolated adult cardiomyocytes from 3 different WT mice were allocated in 5 different groups, being subjected to either normoxic or H/R protocols, in the presence of rMIF or its vehicle (Fig. 4). Analysis of cell death count (expressed as a percentage) within the normoxic groups showed a non-significant increase of cell death after the use of a very high-dose of rMIF (normoxic control of 24.0 ± 4.3 % vs MIF 1 μg/mL of 33.7 ± 5.6 %, *P* = 0.610). Accordingly, rMIF administered before or after an H/R insult resulted in a non-significant increase of cell death compared to those observed in the control group (control: 39.3 ± 3.9 %; rMIF before H/R: 47.6 ± 3.7 %, *P* = 0.794; and rMIF after H/R: 55.5 ± 6.9 %, *P* = 0.134).

### rMIF and the RISK Pathway

**Fig. 5:**
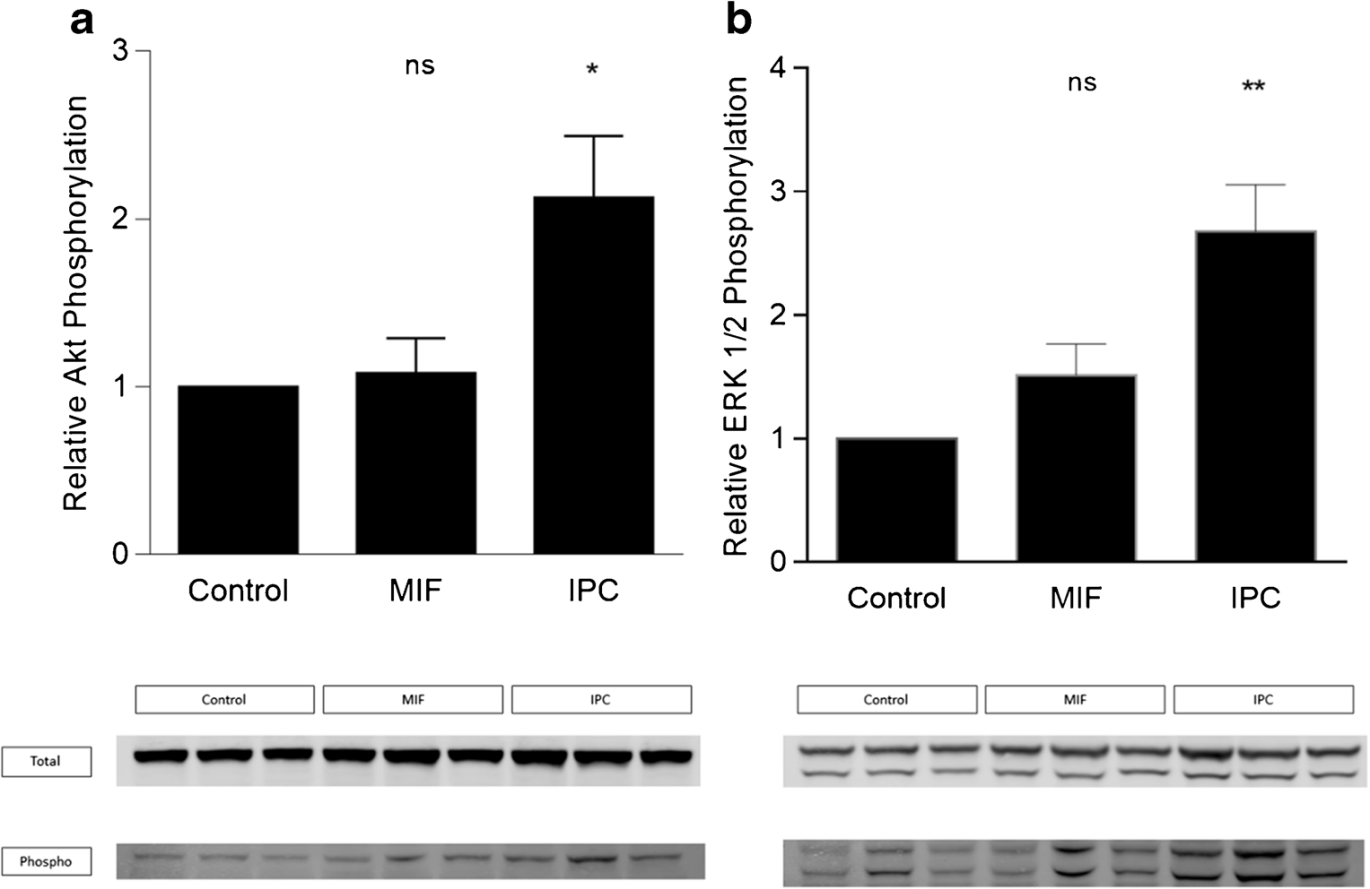
Effect of exogenous rMIF administration on Akt and ERK 1/2 phosphorylation Bar graph showing the percentage of phosphorylation increased in all groups compared to the control group, expressed as mean ± SEM (% of relative protein phosphorylation, normalized by its total), *n* = 3 mice per group. **a**. Phospho-serine 473 Akt levels detected after 15 min of MIF 50 ng/mL perfusion were increased in IPC compared to control group (*P = 0.046), but no effect was detected on Akt phosphorylation in MIF group (*P* = 0. 967). **b**. Phospho-Thr202/Tyr204 ERK 1/2was increased in IPC compared to control group (**P = 0.008), but no effect was detected on ERK 1/2 phosphorylation in MIF group (*P* = 0.358). Abbreviations: IPC, ischemic preconditioning; MIF, macrophage migration inhibitory factor

To assess the effect of rMIF treatment in mouse hearts, Akt and ERK 1/2 phosphorylation were measured following its administration through the ex vivo isolated Langendorff-perfused mouse heart. The failure to reduce myocardial IS was mirrored by an absence of increase in the phosphorylation of both proteins. Considering the control group as a reference category, Akt phosphorylation was increased 1.08 ± 0.21 -fold in the MIF 50 ng/mL group (*P* = 0.967) and 2.13 ± 0.37-fold incremented in the IPC group (*P* = 0.046), whilst in the ERK 1/2 phosphorylation case, the higher dose of MIF showed an increased to 1.52 ± 0.25 -fold (*P* = 0.358) and the IPC to 2.68 ± 0.38 -fold (*P* = 0.008), as illustrated in Fig. 5a and b, respectively.

## Discussion

In this study, the exogenous administration of physiological concentrations of MIF in the 1–50 ng/mL range both before ischemia and at reperfusion failed to reduce IS in an ex vivo perfused murine model of IRI. Moreover, the application of a higher dose in primary isolated cardiomyocytes did not have any statistically significant impact on cell death. Finally, the RISK signalling pathway was not activated when MIF was administered in isolated-perfused hearts, consistent with the absence of any IS-limiting effects. To our knowledge, this is the first study demonstrating that the exogenous administration of MIF fails to confer cardioprotection in WT mice.

Previous evidence suggests that secreted myocardial MIF release in response to I/R, acting in an autocrine/paracrine fashion, can reduce the injury caused by an ischemic insult followed by reperfusion [4, 8, 9]. However, in our isolated mouse heart model, exogenous rMIF administration did not afford the expected cardioprotection. Our seemingly contradictory results did not contravene the previous ones and might be explained by several reasons.

First, in terms of the source of MIF, prior studies have assessed the involvement of endogenous MIF in cardioprotection by suppressing its expression in KO models. These studies have reported an increased damage in those genetic MIF deficiency hearts (*Mif*−/−), whilst MIF restoration abolished this deleterious effect [4, 8, 9].

Only one study reported the effect of MIF in old WT mice, demonstrating that the exogenous MIF administration reduced myocardial IS by the restoration of the impaired AMPK signaling in these aged hearts [22]. Our study offers novel translational insights on the cardioprotective effect of MIF in non-aged WT mice, showing that different doses of MIF did not contribute to reduce IS. Noteworthy, the low-dose of MIF resulted in a non-significant reduction when applied before the ischemic insult, without achieving statistical relevance. Hence, further studies exploring even lower doses might be needed to elucidate if other doses might confer cardioprotection. However, given that the typical measurement of blood MIF levels in AMI patients is in the order of 1.5–30 ng/mL [7], the clinical significance of any observed protection at levels below this would be uncertain. Alternatively, the impact of blocking endogenous MIF in an IPC model could be tested. The recombinant bacterial MIF tested in our study does not have these modifications and may thus not have fully covered the potency of MIF released in an IPC setting. Future experiments could address such a possibility by blocking MIF following cycles of IPC, e.g. with a neutralizing antibody administered after the IPC cycles.

Second, in terms of the experimental protocol, the duration of both ischemia and reperfusion influences the extent of tissue injury [18, 25]. The cardioprotection conferred by MIF has been reported in the specific experimental setting of short IRI (usually 15–20 min of ischemia) [4]. When the period of ischemia has been extended to 30 min followed by a longer reperfusion, the IS-limiting effect of MIF has been lost [9, 18], suggesting that the protection afforded by MIF might be also dependent of the extent of ischemia. Moreover, other authors have speculated that there is a dual-effect of MIF release following an AMI [6], suggesting that an early rise of cardiac-derived MIF (first MIF wave) might be responsible for exerting cardioprotection in the setting of a brief ischemia, followed by an activation of circulating leukocytes under longer ischemic insults, resulting in an increased expression of MIF (second MIF wave) and other inflammatory molecules, that enhance deleterious inflammatory responses [29]. This hypothesis might be linked to other recent publications showing that IRI damage progresses dynamically over time, following a bimodal pattern [30, 31].

Third, the doses were chosen based on MIF plasma levels [7, 17], as well as on those previously used by other research groups [8, 22]. However, it might be possible that the appropriate dose was not used. Prior publications showed a concentration of 20 μg/ml MIF to be cardio-depressant in the heart [9, 20, 21] and a dose of 1 μg/mL to restore increased IS following short IRI damage [10]. However, the results obtained using the ex vivo isolated heart model have been confirmed by the application of a higher dose of MIF to isolated cells. Our viability studies clearly show that such a high dose of rMIF did not increase cell survival. Finally, whether the RISK pathway was activated after MIF administration was also tested. Although Akt and ERK1/2 are not the only pro-survival kinases conferring cardioprotection, whether they were activated by MIF is an important question, given that previous studies have suggested that these kinases are implicated in other pathological processes involving MIF [11–13]. Our results demonstrate that a lack of significant phosphorylation of Akt and ERK1/2 is consistent with the lack of cardioprotection, as well as with a previous study showing that Akt was similar in WT and MIF KO hearts after IRI [8]. However, the activation of other pro-survival kinases also involved in cardioprotection such as AMPK, cannot be ruled out.

Several pre-clinical studies have reported a protective effect of endogenous MIF through the activation of a metabolic pathway [4], the inhibition of pro-apoptotic JNK signalling [8] and the oxidative stress reduction [9], thereby preventing death following brief periods of myocardial IRI. Our results did not challenge the fact that MIF mediates endogenous cardioprotection in a first MIF wave [32], but reinforces the idea that exogenous MIF (either from other cells in a second MIF wave or from an exogenous administration) is not beneficial in terms of IS reduction. However, we should not rule out that the lack of free fatty acids in our Krebs-modified buffer would have an impact in our results, since there is strong evidence that MIF activates AMPK, which in turn increases glucose uptake and glycolysis during ischemia and also enhances fatty acid oxidation during reperfusion.

Despite the fact that no significant differences were found on IS reduction after the use of rMIF at the doses studied, there may still be room for translation. Further studies are needed, particularly using in vivo models to confirm our results as well as using MIF agonists. For example, the cardioprotective role of endogenous MIF following IPC might be increased by MIF agonists, such as MIF20 [15]. In addition, plasma MIF levels are elevated in AMI patients and its use as a prognostic biomarker has been studied in preliminary studies, indicating a predictive value for both IS and cardiac remodelling [17]. Finally, a specific pool of patients might benefit in the future of the potential exogenous administration of MIF, such as elderly patients with an impaired MIF-AMPK activation response [22] and those with a low-expression 5/5 *MIF* promoter genotype and susceptibility to ischemic tissue damage [15]. More attention should be paid to D-dopachrome tautomerase, an enzyme that shares partial sequence and structural homology with MIF, but which lacks certain pro-inflammatory and negative inotropic features and has already demonstrated cardioprotective properties [33].

## Conclusions

Exogenous administration of rMIF both before ischemia and at reperfusion in the doses studied failed to show cardioprotective effects in an ex vivo perfused murine model of IRI, as well as in primary isolated cardiomyocytes. Consistently, the RISK signalling pathway was not activated by rMIF. Although these results do not address the role of endogenous MIF after an ischemic insult followed by reperfusion, they may limit the potential translational value of rMIF.
